# Inotropes and mortality in patients with cardiogenic shock: an instrumental variable analysis from the SWEDEHEART registry

**DOI:** 10.1093/ehjcvp/pvae078

**Published:** 2024-10-16

**Authors:** Petur Petursson, Thorsteinn Gudmundsson, Truls Råmunddal, Oskar Angerås, Araz Rawshani, Moman A Mohammad, Jonas Persson, Joakim Alfredsson, Robin Hofmann, Tomas Jernberg, Ole Fröbert, David Erlinge, Björn Redfors, Elmir Omerovic

**Affiliations:** Department of Cardiology, Institute of Medicine, Department of Molecular and Clinical Medicine, Sahlgrenska University Hospital, Sahlgrenska Academy at University of Gothenburg, Bruna stråket 16, 41345 Gothenburg, Sweden; Department of Cardiology, Institute of Medicine, Department of Molecular and Clinical Medicine, Sahlgrenska University Hospital, Sahlgrenska Academy at University of Gothenburg, Bruna stråket 16, 41345 Gothenburg, Sweden; Department of Cardiology, Institute of Medicine, Department of Molecular and Clinical Medicine, Sahlgrenska University Hospital, Sahlgrenska Academy at University of Gothenburg, Bruna stråket 16, 41345 Gothenburg, Sweden; Department of Cardiology, Institute of Medicine, Department of Molecular and Clinical Medicine, Sahlgrenska University Hospital, Sahlgrenska Academy at University of Gothenburg, Bruna stråket 16, 41345 Gothenburg, Sweden; Department of Cardiology, Institute of Medicine, Department of Molecular and Clinical Medicine, Sahlgrenska University Hospital, Sahlgrenska Academy at University of Gothenburg, Bruna stråket 16, 41345 Gothenburg, Sweden; Department of Cardiology, Institute of Medicine, Department of Molecular and Clinical Medicine, Sahlgrenska University Hospital, Sahlgrenska Academy at University of Gothenburg, Bruna stråket 16, 41345 Gothenburg, Sweden; Department of Cardiology, Institute of Medicine, Department of Molecular and Clinical Medicine, Sahlgrenska University Hospital, Sahlgrenska Academy at University of Gothenburg, Bruna stråket 16, 41345 Gothenburg, Sweden; Department of Cardiology, Institute of Medicine, Department of Molecular and Clinical Medicine, Sahlgrenska University Hospital, Sahlgrenska Academy at University of Gothenburg, Bruna stråket 16, 41345 Gothenburg, Sweden; Department of Cardiology, Institute of Medicine, Department of Molecular and Clinical Medicine, Sahlgrenska University Hospital, Sahlgrenska Academy at University of Gothenburg, Bruna stråket 16, 41345 Gothenburg, Sweden; Department of Cardiology, Institute of Medicine, Department of Molecular and Clinical Medicine, Sahlgrenska University Hospital, Sahlgrenska Academy at University of Gothenburg, Bruna stråket 16, 41345 Gothenburg, Sweden; Department of Cardiology, Institute of Medicine, Department of Molecular and Clinical Medicine, Sahlgrenska University Hospital, Sahlgrenska Academy at University of Gothenburg, Bruna stråket 16, 41345 Gothenburg, Sweden; Department of Cardiology, Institute of Medicine, Department of Molecular and Clinical Medicine, Sahlgrenska University Hospital, Sahlgrenska Academy at University of Gothenburg, Bruna stråket 16, 41345 Gothenburg, Sweden; Department of Cardiology, Institute of Medicine, Department of Molecular and Clinical Medicine, Sahlgrenska University Hospital, Sahlgrenska Academy at University of Gothenburg, Bruna stråket 16, 41345 Gothenburg, Sweden; Department of Cardiology, Institute of Medicine, Department of Molecular and Clinical Medicine, Sahlgrenska University Hospital, Sahlgrenska Academy at University of Gothenburg, Bruna stråket 16, 41345 Gothenburg, Sweden

**Keywords:** Cardiogenic shock, Inotropes, Mortality

## Abstract

**Background:**

The use of inotropic agents in treating cardiogenic shock (CS) remains controversial. This study investigates the effect of inotropes on 30-day mortality in CS patients using data from the SWEDEHEART registry (The Swedish Web-system for Enhancement and Development of Evidence-based care in Heart disease Evaluated According to Recommended Therapies).

**Methods and results:**

Data were sourced from the national SWEDEHEART registry for all CS patients in Sweden from 2000 to 2022. The primary endpoint was 30-day all-cause mortality. We employed multilevel Cox proportional-hazards regression with instrumental variable and inverse probability weighting propensity score to adjust for confounders. The treatment-preference instrument was the quintile of preference for inotrope use at the treating hospital. A total of 16 214 patients (60.5% men, 39.5% women) were included; 23.5% had diabetes, 10.2% had a previous myocardial infarction (MI), and 13.8% had previous heart failure (HF). The median age was 70 years [interquartile range (IQR); 19], with 66.4% over 70. Acute coronary syndrome (ACS) caused CS in 82.9%. Inotropes were administered to 43.8% of patients, while 56.2% did not receive them. There were 7875 (48.1%) deaths. Patients treated with inotropes were, on average, 2 years younger and more likely to have ACS, while those not treated had more previous MI and were less likely to undergo percutaneous coronary intervention (PCI). The number of CS cases decreased by 12% per year (*P*_trend_ < 0.001), and inotrope use increased by 5% per year (*P*_trend_ < 0.001). Unadjusted mortality in CS rose by 2% per calendar year (*P*_trend_ < 0.001). Inotropes were associated with higher mortality [adjusted hazard ratio (HR) 1.72; 95% CI 1.26–2.35; *P* = 0.001], with significant interactions between inotrope treatment, age, and diagnosis (*P*_interaction_ < 0.001 and *P*_interaction_ = 0.018).

**Conclusion:**

In this observational study, inotropes were linked to higher mortality in CS patients, particularly those younger than 70. While CS cases decreased, inotrope use and mortality increased in Sweden.

## Introduction

Cardiogenic shock (CS) is a severe manifestation of heart failure that can occur due to various cardiac conditions, including acute myocardial infarction, severe valvular disease, and cardiomyopathies.^1–4^ Despite advances in managing various cardiac disorders, the CS mortality rate remains high, ranging from 40% to 50%.^5–7^ The mortality in CS has remained high over the last few decades. Inotropic agents are commonly administered in CS to improve cardiac output and perfusion.^8^ However, their use remains controversial due to concerns about their potential adverse effects, including arrhythmias, myocardial ischaemia, and increased mortality.^7^ This study aimed to investigate the effect of treatment with inotropes on mortality in patients with CS using data from the Swedish Web-system for Enhancement and Development of Evidence-based care in Heart disease Evaluated According to Recommended Therapies (SWEDEHEART) registry.^9^

## Methods

### Study design and data source

**Figure 1:**
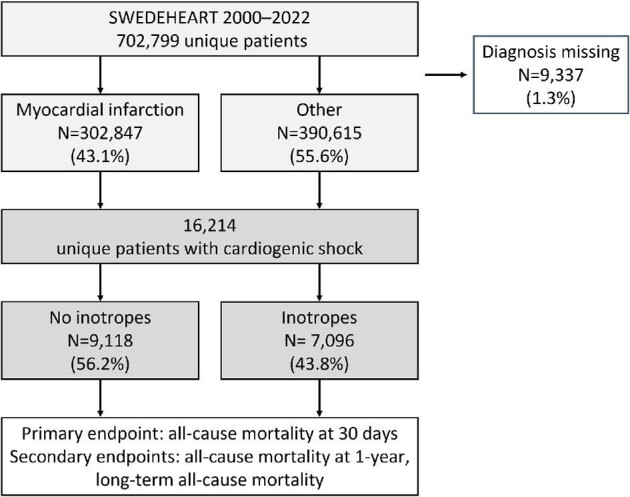
The figure illustrates the patient selection process from the SWEDEHEART registry between 2000 and 2022. Of 702 799 unique patients, 9337 (1.3%) had missing diagnoses. Among the remaining patients, 302 847 (43.1%) were diagnosed with myocardial infarction, and 390 615 (55.6%) had other diagnoses (unstable angina, stable angina, observation for chest pain, myocardial infarct complications, heart failure, arrhythmias, valve disease, myocarditis, cardiomyopathies). Among these, 16 214 unique patients were identified with cardiogenic shock. Subsequent treatment categorization revealed that 9118 patients (56.2%) did not receive inotropic agents, while 7096 patients (43.8%) were treated with inotropic agents.

We conducted an observational study including all patients diagnosed with CS between 2000 and 2022 registered in the SWEDEHEART registry^9^ (*Figure 1*).

### Study population and variables

We identified patients with CS treated in cardiac care units using the International Classification of Diseases, Tenth Revision (ICD-10) codes I21.0 and I50.1. The primary exposure of interest was inotrope use, defined as the administration of noradrenaline, dobutamine, dopamine, milrinone, or any other inotropic agent during hospitalization for CS. CS was defined as SBP less than 90 mmHg or need of vasopressor therapy to achieve a blood pressure at least 90 mmHg; pulmonary congestion or elevated left-ventricular filling pressures; signs of impaired organ perfusion in a normovolaemia or hypervolaemia state, with at least one of the following criteria: altered mental status, cold, clammy skin; oliguria; and increased serum lactate. The primary outcome was 30-day all-cause mortality. The registry collects data on demographic characteristics, comorbidities, clinical presentation, hospital characteristics, and treatment modalities.

### Statistical analysis

We used Cox proportional-hazards regression models adjusted for confounders. To apply a conventional adjustment for selection bias attributable to measured confounding, a multivariable Cox proportional hazards regression was performed on the overall cohort using the following covariates: age at admission, sex, smoking status, history of stroke, chronic kidney disease, history of chronic obstructive pulmonary disease, dementia, heart failure, history of myocardial infarction, diabetes mellitus, peripheral arterial disease, cancer, chronic dialysis treatment, hypertension, coronary artery bypass grafting, percutaneous coronary intervention, diagnosis, coronary angiography, and body mass index. We treated hospitals as a random effect to adjust for patient-clustering within the hospitals.

To account for measurable and unmeasured confounders, we performed instrumental variable (IV) analysis based on the two-stage residual inclusion estimate Cox proportional-hazards regression.^10,11^ IV analysis is a statistical method for the causal inference of an exposure or treatment on an outcome when potentially confounding factors or unmeasured variables might bias the outcome.^12^ It involves IV, a variable that influences the treatment/exposure but is independent of the outcome. Typically, a two-stage regression model is utilized for IV analysis implementation. Initially, a regression model is fitted with the IV as the predictor variable and the treatment/exposure variable as the outcome variable. In the second stage, the predicted values of the treatment/exposure are used as the independent variable in another regression model. The outcome variable is regressed on the predicted values of the treatment/exposure while adjusting for potential confounding variables. In our investigation, the IV utilized was the quintile of preference for administering inotropes at the treating hospitals. We did subgroup analyses to see how age, gender, diabetes, and type of hospital modified the observed effects.

Every analysis adhered to the accepted definition of statistical significance, which is a two-tailed *α* = 0.05. Version 4.3.0 R (Foundation for Statistical Computing) was used for all statistical calculations and data visualization. The lmtest and ivtools packages were used for two-stage residual inclusion analysis and IV validity testing. The survival and survminer packages were used for survival analyses, the gtsummary package for creating tables, and the forestploter package for creating forest plots.

Missing values in the dataset were imputed using the missRanger package in R, which utilizes random forest imputation to fill in missing values.^13^ The missRanger method has been shown to produce accurate imputations while preserving the data distribution, making it a reliable approach to missing data imputation.^13^

We performed sensitivity analyses to assess our findings' robustness and the potential impact of misclassification and unmeasured confounding.^14,15^ For this purpose, we utilized two R packages: tipr and episensr.

## Results

### Study population

We included 16 214 patients with CS in the analysis (*Figure 1*). *Table 1* summarizes patients' characteristics, and *Table 2* presents medication at admission. Supplementary material online, Table S1 presents a summary of patient characteristics according to the quintiles of the hospital's preference for inotrope treatment.

**Table 1:** Patient's characteristics

Variable	*N*	No inotropes *N* = 9118^a^	Inotropes *N* = 7096^a^	Difference^b^	95% CI^b,c^
Age	16 214	76 (66, 83)	74 (65, 80)	0.14	0.11, 0.17
Male sex	16 214	5315 (58%)	4511 (64%)	0.11	0.08, 0.14
Diabetes	14 982	1670 (19%)	1491 (23%)	0.10	0.06, 0.13
Smoking	15 308			0.16	0.13, 0.19
*No*		3843 (45%)	2647 (39%)		
*Yes*		2021 (24%)	1522 (23%)		
*Previous*		1691 (20%)	1521 (23%)		
Stroke	16 214	1165 (13%)	814 (11%)	0.04	0.01, 0.07
Renal failure	16 214	328 (3.6%)	274 (3.9%)	0.01	−0.02, 0.04
COPD	16 214	683 (7.5%)	537 (7.6%)	0.00	−0.03, 0.03
Dementia	16 214	80 (0.9%)	36 (0.5%)	0.04	0.01, 0.08
Heart failure	16 214	1378 (15%)	863 (12%)	0.09	0.06, 0.12
Myocardial infarction	16 214	985 (11%)	675 (9.5%)	0.04	0.01, 0.07
PAD	16 214	596 (6.5%)	437 (6.2%)	0.02	−0.02, 0.05
Cancer	16 214	443 (4.9%)	291 (4.1%)	0.04	0.01, 0.07
Dialysis	16 214	77 (0.8%)	57 (0.8%)	0.00	−0.03, 0.04
Hypertension	16 214	2172 (24%)	1690 (24%)	0.00	−0.03, 0.03
Previous CABG	16 214	285 (3.1%)	241 (3.4%)	0.02	−0.02, 0.05
Previous PCI	16 214	120 (1.3%)	103 (1.5%)	0.01	−0.02, 0.04
CPR	16 214	690 (7.6%)	1157 (16%)	−0.27	−0.30, −0.24
IABP	16 214	191 (2.1%)	625 (8.8%)	−0.30	−0.33, −0.27
ECMO/Impella	16 214	20 (0.2%)	80 (1.1%)	−0.11	−0.14, −0.08
CPAP	16 214	1235 (14%)	2089 (29%)	0.39	0.36, 0.43
PM/ICD	16 214			0.04	0.01, 0.07
*No device*		8865 (97%)	6856 (97%)		
*Pacemaker*		151 (1.7%)	136 (1.9%)		
*ICD*		86 (0.9%)	88 (1.2%)		
*PM + ICD*		3 (<0.1%)	2 (<0.1%)		
*CRT*		4 (<0.1%)	5 (<0.1%)		
*ICD + CRT*		9 (<0.1%)	9 (0.1%)		
Diagnosis	16 085			0.31	0.28, 0.34
*MI*		6617 (73%)	5971 (85%)		
*UA*		207 (2.3%)	63 (0.9%)		
*SA*		377 (4.2%)	161 (2.3%)		
*HF*		515 (5.7%)	277 (3.9%)		
*Arrhythmia*		532 (5.9%)	273 (3.9%)		
*Other*		*812 (8.9%)*	*284 (4.0%)*		
Angiography	16 214	3652 (40%)	4252 (60%)	0.41	0.37, 0.44
PCI performed	16 214	2839 (31%)	3643 (51%)	0.42	0.39, 0.45

COPD, chronic obstructive lung disease; PAD, peripheral artery disease; CABG, coronary artery by-pass surgery; PCI, percutaneous coronary intervention; MI, myocardial infarction; UA, unstable angina; SA, stable angina; HF, heart failure; IABP, intra-aortic balloon pump; ECMO, extracorporeal membrane oxygenation; PM, pacemaker; ICD, implantable cardioverter-defibrillator; CRT, cardiac resynchronization therapy; CPAP, continuous positive airway pressure; and CPR, cardiopulmonary resuscitation (before hospitalization).

^a^ *n* (%); Median (IQR).

^b^Standardized mean difference.

^c^CI, confidence interval.

**Table 2:** Medication at admission

Variable	*N*	No inotropes *N* = 9118^a^	Inotropes *N* = 7096^a^	Difference^b^	95% CI^b,c^
ACEI	16 036	1806 (20%)	1380 (20%)	0.10	0.07, 0.13
ARB	9166	595 (13%)	601 (13%)	0.06	0.02, 0.10
Anticoagulants	16030			0.09	0.06, 0.12
No		8194 (91%)	6301 (90%)		
Warfarin		551 (6.1%)	404 (5.8%)		
Dabigatran		10 (0.1%)	14 (0.2%)		
Rivaroxaban		20 (0.2%)	17 (0.2%)		
Apixaban		59 (0.7%)	75 (1.1%)		
Other		5 (<0.1%)	2 (<0.1%)		
Aspirin	16 045	2922 (32%)	1991 (28%)	0.11	0.08, 0.14
Beta-blocker	16 033	2877 (32%)	2164 (31%)	0.09	0.06, 0.12
Ca^+^ antagonist	16 008	1382 (15%)	1125 (16%)	0.10	0.06, 0.13
Digitalis	16 023	610 (6.8%)	327 (4.7%)	0.11	0.08, 0.14
Diuretic	16 035	2797 (31%)	1901 (27%)	0.12	0.09, 0.15
Statin	16 026	1409 (16%)	1233 (18%)	0.10	0.07, 0.13
Ezetimibe	15 275	30 (0.4%)	33 (0.5%)	0.08	0.04, 0.11
Nitrate	16 020	973 (11%)	556 (7.9%)	0.12	0.09, 0.15

ACE, angiotensin-converting enzyme inhibitor; ARB, angiotensin II receptor blocker.

^a^ *n* (%).

^b^Standardized mean difference.

^c^CI, confidence interval.

**Figure 2:**
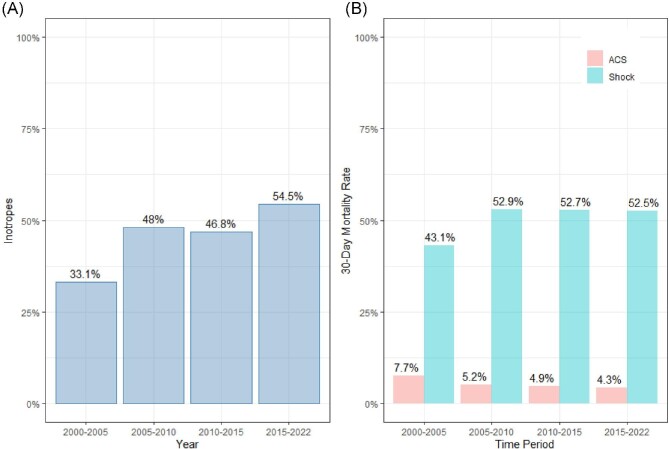
Trends in utilization of inotropes and mortality in Sweden 2000–22. Panel A shows the average use of inotropes over time. The *y*-axis shows the proportion of patients who received inotropes, while the *x*-axis displays the different periods. Inotropes increased over time, with the highest use during the last period. Panel B depicts the 30-day mortality rate over time, stratified by shock status and ACS diagnosis. The *y*-axis shows the proportion of patients who died within 30 days of hospital admission, while the *x*-axis displays the different periods. The figure demonstrates that the mortality rate decreased over time in patients with acute coronary syndrome (ACS) but remained high in patients with shock.

Generally, a standardized mean difference (SMD) of 0.15 or lower is considered small. The median age was 70 years (interquartile range; 19), and 66.4% were >70. The most common cause of CS was acute coronary syndrome (ACS) (82.9%). Inotropic agents were used in 43.8% of patients, while 56.2% did not receive inotropic agents. The only variables with SMD >0.1 between the two groups were age, sex, smoking, diagnosis, angiography, and PCI. On average, patients treated with inotropes were 2 years younger and more likely to have ACS. Patients not treated with inotropes were less likely to undergo angiography and PCI and were more likely to be smokers. There were no substantial differences in drugs at the time of admission between the two groups. The number of patients with CS decreased by 12% per calendar year (*P*_trend_ < 0.001). The use of inotropes increased by 5% per calendar year (*P*_trend_ < 0.001, *Figure 2*A).

### Inotrope use and mortality

**Figure 3:**
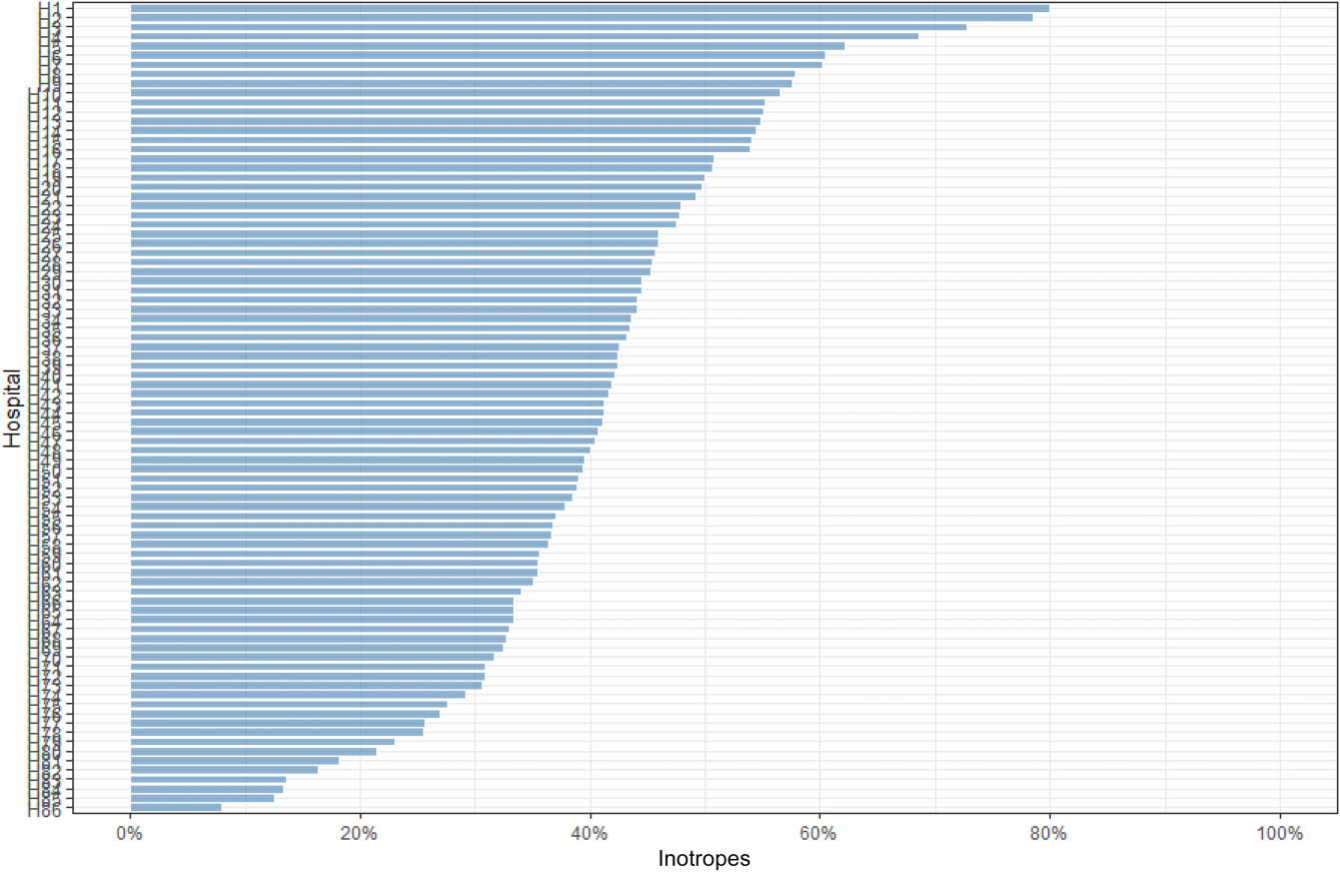
The difference in utilization of inotropes among hospitals in Sweden. The figure illustrates the considerable variation in the preference for using inotropes across different hospitals. The data shows that the use of inotropes varied significantly, ranging from 25% in some hospitals to as high as 78% in others. This considerable variation indicates a lack of standardization in the use of inotropes for managing patients with cardiogenic shock in hospitals.

**Figure 4:**
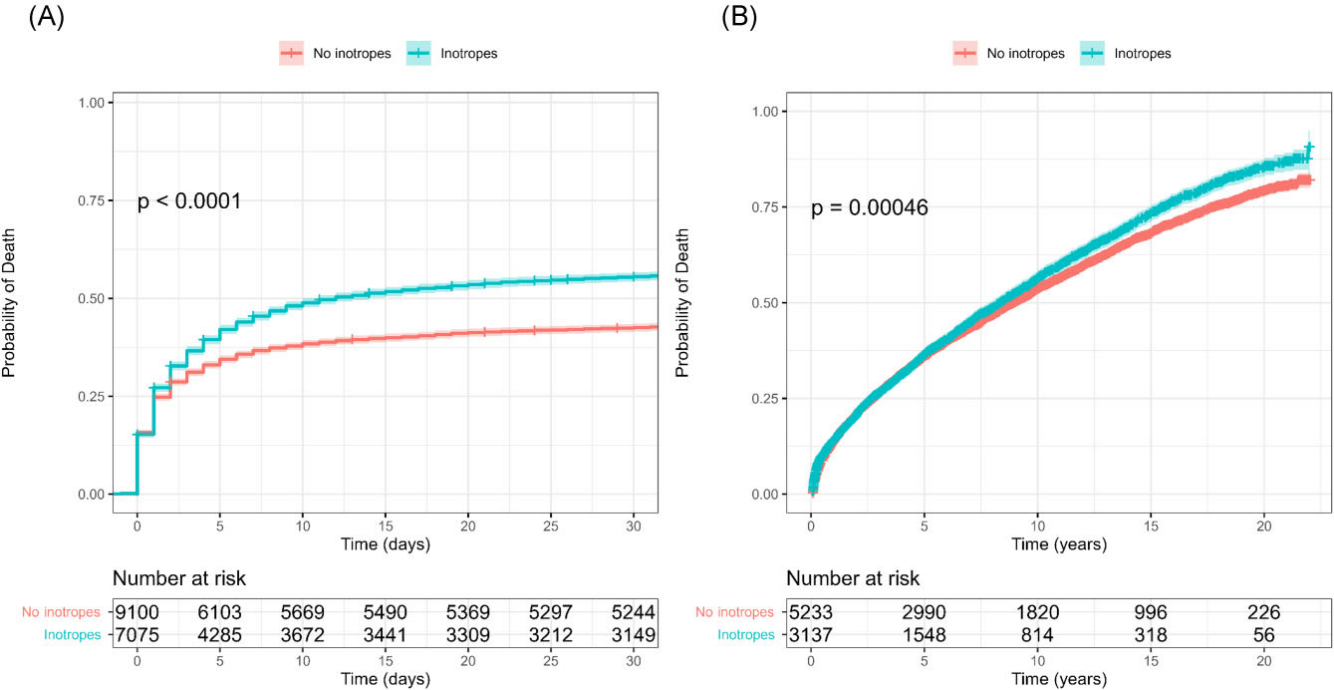
Kaplan-Meier curves for all-cause mortality in patients with cardiogenic shock. Panel A compares 30-day all-cause mortality in patients treated with inotropes vs. those not. The log-rank test in Panel A demonstrates a significant difference in mortality rates between the two groups (*P* < 0.001). The risk table below Panel A displays the number of patients at risk every 5 days for each group. Panel B shows survival after 30 days for the same groups as in Panel A.

The unadjusted mortality rate in patients with CS increased by 2% per calendar year (*P*_trend_ < 0.001). While 30-day mortality decreased in patients with ACS (*P*_trend_ < 0.001), CS mortality increased after 2005 (*P* < 0.001, *Figure 2*B). There was a considerable variation between hospitals in preference for using inotropes, ranging from 8% to 80% (*P*_trend_ < 0.001, *Figure 3*). The mean follow-up time was 3.8 years (range 0–22 years). Unadjusted 30-day (*P* < 0.001, *Figure 4*A) and long-term mortality (*P* = 0.00031, *Figure 4*B) were higher in the inotrope group.

#### Instrumental variable analysis

In the first stage of our IV analysis, we identified a significant association between the treatment preference instrument and inotrope use in CS patients (*P* < 0.001). In the second stage of the analysis, we observed a 72% increase in 30-day mortality linked to inotrope use (*Table 3*, *P* < 0.001). Our instrument passed the under-identification test (*P* < 0.001), suggesting it was relevant. The overidentification test (*P* = 0.425) did not reject the null hypothesis, suggesting that our instrument was valid. The instrument also passed the weak identification test (Cragg-Donald Wald F statistic = 150, *P* < 0.001), demonstrating a strong correlation between the instrument and the treatment.

**Table 3:** Risk estimates in different statistical models

Model	HR	95% CI	*P*-value
30-days mortality			
Unadjusted Cox regression	1.62	1.53–1.71	<0.001
Multivariable Cox regression^a^	1,81	1.71–1.92	<0.001
IV Cox regression^b^	1.72	1.26–2.35	0.001
1-year mortality			
Unadjusted Cox regression	1.46	1.39–1.53	<0.001
Multivariable Cox regression^a^	1.69	1.60–1.79	<0.001
IV Cox regression^b^	1.41	1.06–1.87	0.018
Long-term mortality			
Unadjusted Cox regression	1.35	1.30–1.40	<0.001
Multivariable Cox regression^a^	1.48	1.42–1.54	<0.001
IV Cox regression^b^	1.20	0.96–1.51	0.099

a Multivariable proportional hazards Cox regression with the following covariates: age at admission, sex, smoking status, history of stroke, chronic kidney disease, history of chronic obstructive pulmonary disease, dementia, heart failure, history of myocardial infarction, diabetes mellitus, peripheral arterial disease, cancer, chronic dialysis treatment, hypertension, coronary artery bypass grafting, percutaneous coronary intervention, diagnosis, coronary angiography, and body mass index.

b Instrumental variable (IV) analysis with treatment-preference instrument based on the quintiles of the hospital preference for using inotropes for treating patients with cardiogenic shock. The IV model also used all the covariates from the multivariable Cox proportional hazards regression.

#### Multivariable Cox proportional-hazards regression

The risk of death at 30 days was higher in patients treated with inotropes [adjusted hazard ratio (HR_adj_) 1.81; 95% confidence interval (CI) 1.71–1.92; *P* < 0.001, *Figure 4*A]. The risk of death in the long term was also higher in patients treated with inotropes (HR_adj_ 1.48; 95% CI 1.42–1.54; *P* < 0.001, *Figure 4*B).

#### Subgroup analysis

**Figure 5:**
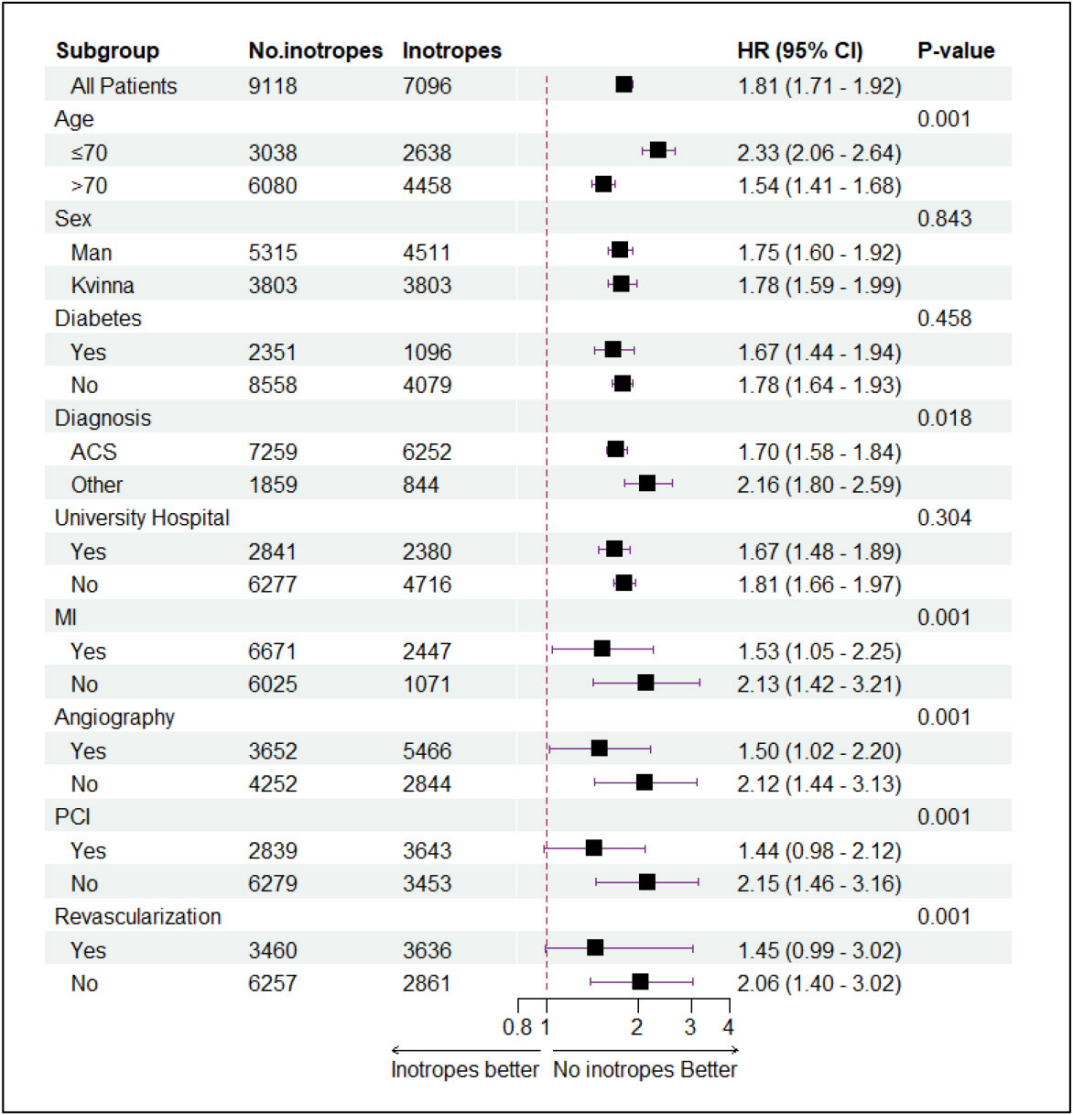
The figure displays a subgroup analysis for age, sex, diabetes, diagnosis, and type of hospital. The data are presented as a forest plot, which summarizes the hazard ratios (HR) and 95% confidence intervals (CI) of the different subgroups on the 30 days mortality. The results of the subgroup analysis indicate that the effect of the inotropes varied across some subgroups. There was evidence for quantitative interaction with a higher risk in younger adults (<70 years) and in patients with cardiogenic shock not caused by acute coronary syndrome (ACS).

We found a significant quantitative interaction between treatment with inotropes and age and the cause of CS (*Figure 5*). Patients >70 years (HR_adj_ 1.54; 95% CI 1.41–1.68; *P*_interaction_ < 0.001) and patients in whom CS was caused by ACS (HR_adj_ 1.70; 95% CI 1.58–1.84; *P*_interaction_ = 0.018) had a lower risk of death when treated with inotropes. There was no interaction between inotropes and gender, diabetes, or hospital type.

#### Sensitivity analysis

One or more unmeasured confounders would nullify the observed OR of 1.72 if: (i) the prevalence of the unmeasured confounder in the exposed population was 60%, (ii) the estimated prevalence of the unmeasured confounder in the unexposed population was 30%, and (iii) an estimated relationship between the unmeasured confounder and the outcome of 9.6 in OR. The misclassification bias for CS diagnosis needed to nullify the OR of 1.72 is 23%. A placebo outcome sensitivity analysis was conducted to validate the instrument using stroke as an unrelated outcome. We found no significant association between inotrope usage and stroke at 30 days (HR 1.0; 95% CI 0.01–198, *P* = 1.00), at 1 year (HR 1.0; 95% CI 0.19–5.10, *P* = 1.00), and long-term (HR 1.0; 95% CI 0.46–2.06, *P* = 1.00). To evaluate whether treatment-induced selection bias^16^ is affecting the IV risk estimates, we applied inverse probability of selection weights.^17^ The estimated risk from the IV model remained substantially unchanged for mortality at 30 days (HR 1.73; 95% CI 1.26–2.37, *P* = 0.001), at 1 year (HR 1.38; 95% CI 1.04–1.84, *P* = 0.027), and long-term (HR 1.19; 95% CI 0.96–1.49, *P* = 0.111). These results support the instrument's validity, implying it is not associated with treatment-induced selection bias.

## Discussion

Our research explored the impact of inotropic therapy on mortality rates, analysing data from a large cohort of 16 214 patients with CS recorded in the SWEDEHEART registry. We found a strong association between inotrope use and elevated mortality rates in patients with CS. This study represents one of the most comprehensive observational investigations into using inotropes in the context of CS.

The use of inotropic agents in the management of patients with CS has been a subject of controversy for several decades.^2,4,18,19^ Inotropes increase myocardial contractility and improve haemodynamics, which makes them an attractive therapeutic option for managing CS.^8^ However, some studies have raised concerns about the safety and efficacy of inotropes, suggesting that they may be associated with an increased mortality risk in patients with CS.^7,20–22^ Our study highlights the risks associated with inotropic therapy and underscores the need for careful consideration when determining appropriate treatment strategies for CS patients. Our findings are consistent with previous studies reporting that inotropes are associated with an increased risk of mortality in CS.^23–26^ One potential explanation for the negative mortality impact is that inotropes increase myocardial oxygen demand, leading to myocardial ischaemia and worsening heart failure with consequent haemodynamic instability.^1,21,27^ In addition, inotropes may trigger malignant ventricular arrhythmias, further deteriorating the clinical course of patients with CS.^6^

Patients who did not receive inotropes were less likely to undergo angiography and PCI, likely reflecting a more conservative management approach. Despite a substantial proportion of ACS diagnoses in the non-inotrope group, the significantly lower procedural rates suggest that factors such as clinical frailty, comorbidities, or higher procedural risk influenced the decision-making process.

The findings of our study indicate a concerning trend in managing patients with cardiogenic CS. Despite advancements in the treatment for ACS over the past two decades, the mortality rate for patients with CS has remained stagnant at approximately 50%. This lack of improvement in mortality for CS patients contrasts with the significant decrease in mortality for patients with ACS during the same period. One possible explanation for these opposite results is the increased utilization of inotropic therapy. We found a significant relationship between the administration of inotropes and increased short- and long-term mortality rates among CS patients. One could assume that inotrope treatment is used in the most haemodynamically unstable patients. However, all patients in the study were diagnosed with CS and were haemodynamically unstable. The substantial variation between hospitals indicates a lack of standardization in the use of inotropes for managing patients with CS. This difference in preference for inotropes among hospitals has created ‘natural randomization,’ which we could utilize for causal statistical interference with instrumental variable analysis.^12^ Several formal tests support the instruments' validity, and the analyses speak against treatment-induced selection bias.^16,17^ Furthermore, the sensitivity analyses demonstrated the IV model's resistance to residual confounding bias. As a result, our study warns against overusing inotropes in treating CS patients and emphasizes the importance of focusing research on alternative treatments that improve survival.

We found that the higher risk of mortality associated with inotrope use was more pronounced in patients younger than 70 years. Various possible explanations exist for the lower risk in elderly CS patients treated with inotropes. Clinicians may adopt a more selective and cautious approach when treating elderly CS patients with inotropes. Older patients may be less likely to receive aggressive interventions, such as mechanical support, which could reduce the risk of complications associated with high-risk procedures. Additionally, the clinical threshold for using inotropes may be higher in older patients, meaning those who do receive them are more carefully selected based on a favourable risk-benefit profile.

The strength of our paper lies in several aspects, including the large number of death events (∼8000), which provides high statistical power. We utilized instrumental variable analysis, considered the gold standard for statistical causal inference from observational data.^12,28,29^ This method allows adjustment for known and unknown confounding factors and mitigates the effects of confounding and selection bias often inherent in observational studies.

### Limitations

We acknowledge the significant limitations inherent in observational studies. Our study's absence of randomization, blinding, and placebo control means it cannot definitively answer whether inotropes are beneficial or harmful in patients with CS. Despite our efforts to adjust for confounders, residual confounding remains a possibility. These are fundamental challenges observational research faces, which is inherently limited in replicating the rigor of randomized controlled trials (RCTs). However, observational studies, including ours, are an essential component of evidence-based medicine, particularly when RCTs are not feasible due to ethical or practical constraints or have yet to be conducted. They offer critical insights into real-world clinical practices and patient outcomes, forming the basis for generating hypotheses and guiding future research. We employed IV analysis to strengthen causal inference, a method widely recognized for mitigating confounding and selection bias in observational data. The variation in hospital preferences for inotrope use is a quasi-randomizing factor, allowing for more robust estimates of causal relationships. While this approach does not fully replicate the rigor of RCT, it offers a well-validated method for causal inference, as highlighted by recent advances in natural experiment methodologies.

The Swedish healthcare system in which our study was conducted may limit the generalizability of our findings to other populations or healthcare systems. A significant limitation of our study is the lack of specific data on shock severity, including critical parameters such as lactate levels, liver function, and the occurrence of cardiac arrest. These variables are not captured in the SWEDEHEART registry, limiting our ability to fully characterize the severity of CS in our patient population. As a result, we could not incorporate these important clinical markers into our regression model. Additionally, the absence of data on complications associated with mechanical circulatory support, such as bleeding, infection, and thromboembolism, may affect the interpretation of mortality outcomes. Another important limitation is the lack of detailed information regarding the specific type, dosage, and duration of inotropic therapy in our dataset. This limitation introduces the potential for confounding, as different inotropes can affect patient outcomes. For instance, milrinone, a phosphodiesterase-3 inhibitor, increases myocardial oxygen demand and may exacerbate ischaemia, particularly in patients with ACS. Although there is a theoretical concern about milrinone's ability to worsen ischaemia due to its vasodilatory effects,^30^ clinical trials have not demonstrated an increased risk of complications such as arrhythmias, worsening heart failure, or death in patients with CS, including those with ACS.^33^ Available meta-analyses have also not shown a clear mortality benefit for one inotrope over another.^31,32^ However, our lack of granular data prevents us from fully exploring the differential effects of inotropes like milrinone, dobutamine, and noradrenaline on specific subgroups, including those with ACS. Additionally, our study does not include cause-specific mortality data, limiting our ability to differentiate between cardiovascular and non-cardiovascular deaths. As a result, the analysis is based on all-cause mortality, which, while comprehensive, may not fully capture the nuances of cardiovascular outcomes. Future research with more detailed inotrope reporting and patient stratification would be valuable in clarifying these outcomes. In addition, our database did not include invasive hemodynamic measures, and in some cases, inotropes may have been administered for palliative care, which could influence mortality outcomes. Despite these limitations, the large cohort size and the use of IV analysis provide a strong analytical framework to explore the association between inotrope use and mortality in CS patients.

Importantly, we recognize that randomized controlled trials are necessary to definitively establish whether inotropes are beneficial, neutral, or harmful in treating CS. An ongoing example is the CAPITAL DOREMI2 trial,^34^ a placebo-controlled RCT investigating milrinone in CS. Such trials will help resolve some of these critical questions and determine the optimal therapy type, dosage, and duration.

In conclusion, our study contributes meaningful insights to the ongoing debate on the role of inotropes in CS management. Still, it should be interpreted cautiously, given the inherent limitations of observational research. Until more definitive evidence emerges from RCTs, we recommend careful consideration of inotropes, using the lowest effective dose for the shortest necessary duration or their avoidance altogether in certain cases.

## Supplementary Material

pvae078_Supplemental_File

## Data Availability

The article's data cannot be shared publicly for ethical and privacy reasons.
