# Supplementary material for: Inotropes and mortality in patients with cardiogenic shock: an instrumental variable analysis from the SWEDEHEART registry
Source: Eur Heart J Cardiovasc Pharmacother. 2024 Oct 16;11(1):57–65. doi: 10.1093/ehjcvp/pvae078 (PMC11805686; doi:10.1093/ehjcvp/pvae078)
Supplement: pvae078_Supplemental_File [file pvae078_supplemental_file.docx]

| **Variable** | **N** | **Quintile I**  N = 3329*^1^* | **Quintile II**  N = 2637*^1^* | **Quintile III**  N = 3613*^1^* | **Quintile IV**  N = 3050*^1^* | **Quintile V**  N = 3585*^1^* |
| --- | --- | --- | --- | --- | --- | --- |
| Death at 30 days | 16,143 | 1,550 (47%) | 1,290 (49%) | 1,734 (48%) | 1,414 (47%) | 1,784 (50%) |
| Death at one year | 16,214 | 1,834 (55%) | 1,495 (57%) | 2,006 (56%) | 1,662 (54%) | 2,002 (56%) |
| Inotropes | 16,214 | 1,044 (31%) | 1,024 (39%) | 1,518 (42%) | 1,392 (46%) | 2,118 (59%) |
| Age | 16,214 | 76 (66, 82) | 75 (66, 83) | 75 (65, 82) | 74 (64, 81) | 74 (64, 81) |
| Male sex | 16,214 | 1,952 (59%) | 1,597 (61%) | 2,167 (60%) | 1,871 (61%) | 2,239 (62%) |
| Smoking | 16,214 |  |  |  |  |  |
| *No* |  | 1,972 (61%) | 1,544 (59%) | 2,106 (58%) | 1,662 (54%) | 2,048 (57%) |
| *Yes* |  | 727 (22%) | 600 (23%) | 798 (22%) | 710 (23%) | 798 (22%) |
| *Previous* |  | 630 (19%) | 493 (19%) | 709 (20%) | 678 (22%) | 739 (21%) |
| Diabetes | 16,214 | 706 (21%) | 536 (20%) | 760 (21%) | 622 (20%) | 759 (21%) |
| Hypertension | 16,214 | 772 (23%) | 606 (23%) | 804 (22%) | 769 (25%) | 911 (25%) |
| Stroke | 16,214 | 427 (13%) | 338 (13%) | 423 (12%) | 349 (11%) | 442 (12%) |
| Renal failure | 16,214 | 120 (3.6%) | 98 (3.7%) | 142 (3.9%) | 110 (3.6%) | 132 (3.7%) |
| COPD | 16,214 | 262 (7.9%) | 196 (7.4%) | 269 (7.4%) | 225 (7.4%) | 268 (7.5%) |
| Dementia | 16,214 | 29 (0.9%) | 15 (0.6%) | 23 (0.6%) | 26 (0.9%) | 23 (0.6%) |
| Heart failure | 16,214 | 542 (16%) | 380 (14%) | 488 (14%) | 384 (13%) | 447 (12%) |
| Myocardial infarction | 16,214 | 338 (10%) | 251 (9.5%) | 379 (10%) | 307 (10%) | 385 (11%) |
| PAD | 16,214 | 217 (6.5%) | 158 (6.0%) | 241 (6.7%) | 186 (6.1%) | 231 (6.4%) |
| Cancer | 16,214 | 132 (4.0%) | 115 (4.4%) | 163 (4.5%) | 153 (5.0%) | 171 (4.8%) |
| Dialysis | 16,214 | 23 (0.7%) | 16 (0.6%) | 30 (0.8%) | 29 (1.0%) | 36 (1.0%) |
| CABG | 16,214 | 108 (3.2%) | 78 (3.0%) | 94 (2.6%) | 117 (3.8%) | 129 (3.6%) |
| PCI | 16,214 | 39 (1.2%) | 35 (1.3%) | 52 (1.4%) | 38 (1.2%) | 59 (1.6%) |
| Diagnosis | 16,214 |  |  |  |  |  |
| *MI* |  | 2,399 (72%) | 1,957 (74%) | 2,922 (81%) | 2,397 (79%) | 3,014 (84%) |
| *UA* |  | 62 (1.9%) | 46 (1.7%) | 58 (1.6%) | 51 (1.7%) | 53 (1.5%) |
| *SA* |  | 140 (4.2%) | 87 (3.3%) | 120 (3.3%) | 100 (3.3%) | 98 (2.7%) |
| *HF* |  | 1 (<0.1%) | 1 (<0.1%) | 3 (<0.1%) | 2 (<0.1%) | 0 (0%) |
| *Arrhythmia* |  | 221 (6.6%) | 156 (5.9%) | 159 (4.4%) | 144 (4.7%) | 119 (3.3%) |
| *Other* |  | 506 (15%) | 390 (14.7%) | 351 (9.7%) | 356 (11.8%) | 301 (8.4%) |
| Angiography | 16,214 | 1,334 (40%) | 1,110 (42%) | 1,658 (46%) | 1,612 (53%) | 2,190 (61%) |
| PCI treatment | 16,214 | 1,044 (31%) | 885 (34%) | 1,401 (39%) | 1,313 (43%) | 1,839 (51%) |
| CPR | 16,214 | 291 (8.7%) | 294 (11%) | 375 (10%) | 364 (12%) | 523 (15%) |
| IABP | 16,214 | 91 (2.7%) | 102 (3.9%) | 136 (3.8%) | 146 (4.8%) | 341 (9.5%) |
| ECMO/Impella | 16,214 | 14 (0.4%) | 9 (0.3%) | 18 (0.5%) | 24 (0.8%) | 35 (1.0%) |
| CPAP | 16,214 | 647 (19%) | 583 (22%) | 597 (17%) | 657 (22%) | 840 (23%) |
| PM/ICD | 16,214 |  |  |  |  |  |
| *No device* |  | 3,234 (97%) | 2,556 (97%) | 3,516 (97%) | 2,935 (96%) | 3,480 (97%) |
| *Pacemaker* |  | 51 (1.5%) | 48 (1.8%) | 54 (1.5%) | 74 (2.4%) | 60 (1.7%) |
| *ICD* |  | 40 (1.2%) | 27 (1.0%) | 34 (0.9%) | 37 (1.2%) | 36 (1.0%) |
| *PM + ICD* |  | 2 (<0.1%) | 1 (<0.1%) | 0 (0%) | 1 (<0.1%) | 1 (<0.1%) |
| *CRT* |  | 1 (<0.1%) | 1 (<0.1%) | 1 (<0.1%) | 2 (<0.1%) | 4 (0.1%) |
| *ICD + CRT* |  | 1 (<0.1%) | 4 (0.2%) | 8 (0.2%) | 1 (<0.1%) | 4 (0.1%) |
| *^1^*n (%); Median (Q1, Q3) | | | | | | |

COPD = chronic obstructive lung disease, PAD = peripheral artery disease, CABG = coronary artery by-pass grafting, PCI = percutaneous coronary intervention, MI = myocardial infarction, UA = unstable angina, SA = stable angina, HF = heart failure, IABP = intra-aortic balloon pump, ECMO = extracorporeal membrane oxygenation, PM = pacemaker, ICD = implantable cardioverter-defibrillator, CRT = cardiac resynchronization therapy, CPAP = continuous positive airway pressure, CPR = cardiopulmonary resuscitation (before hospitalization)
